# Model Construction and Research on Decision Support System for Education Management Based on Data Mining

**DOI:** 10.1155/2021/9056947

**Published:** 2021-12-20

**Authors:** Weidan Wang

**Affiliations:** School of Political and Public Management, Zhengzhou University, Zhengzhou, Henan 450000, China

## Abstract

Based on data mining technology, this paper applies a combination of theoretical and practical approaches to systematically describe the background and basic concepts related to the generation of data mining-related technologies. The classical data mining process is analyzed in depth and in detail, and the method of building a decision support system for education management based on the B/S model is studied. Not only are the data mining techniques applied to this system, but also the decision tree model with the improved ID3 algorithm is implemented in this thesis, which is further applied to the educational management decision support system of this topic. The load of the client computer is reduced, and the client computer only needs to run a small part of the program. This paper focuses on the following aspects: the overall planning of the educational management decision support system based on data mining technology. From the actual educational management work, we analyze the requirements and design each functional module of this system in detail, applying the system functional structure diagram and functional use case diagram to represent the functional structure of the system and using flow charts to illustrate the workflow of the system as a whole and in parts. The logical structure design, entity-relationship design, and physical model design of the database have been carried out. To improve the efficiency of the system, the ID3 algorithm was improved on this basis to reduce the time complexity of its operation, improve the efficiency of the system operation, and achieve the goal of assessing and predicting the teaching quality of teachers. The development and design of this system provide an efficient, convenient, scientific, and reliable system tool to reduce the workload of education administrators and, more importantly, to make reasonable and effective use of the large amount of data generated in the management, and data mining techniques are used to extract valuable and potential information from these data, which can be more scientific and efficient for the teaching of teachers and students. It can provide reliable, referenceable, and valuable information for managers to make assessments and decisions.

## 1. Introduction

In the current context of rapid economic growth and rising urbanization, education resources, as one of the important social public resources, are lagging in terms of technical means of management, which poses a negative impact on the deepening of education reform and the optimal allocation of education resources, thus making the development of these tasks face serious challenges [1]. With the continuous progress of information technology, information technology has been widely used in the field of education management at home and abroad to improve their management level [2]. Although the educational information management systems currently in use are also based on database construction, such systems can only achieve simple analysis and management of data, and the decision-making function is relatively weak. The system can collect education-related data and conduct simple analysis and statistics on the collected data, but its functions are relatively weak or even absent when it comes to the comprehensive analysis of education management. Many developed countries in the world have established a complete, high-tech, and scientific education management decision support system, which has a huge guiding role in the economic development of their national systems. Many colleges and universities have also established educational management decision support systems that are suitable for their own development in local area networks and wide area networks [3]. At the same time, the use of data mining technology in education management decision-making has also received more and more attention from universities, and better application of data mining to provide services for education management and decision-making has become a new issue facing education [4].

For quite a long time in the past, educational decision-making was based on leadership intuition as well as relevant experience or even relied on social trends, which is obviously not in line with scientific decision theory, and therefore became an important criticism content for educational research staff. Scientific decision-making is inseparable from the necessary data support, but, for a long time in the field of education, a lot of data helpful for decision-making is often scattered on the desks and beds of faculty members [5]. Therefore, strengthening the construction of information technology systems, centralizing and unifying the management of large amounts of data, and then using the corresponding algorithms to analyze them will enable decision-making managers to quickly obtain a large amount of data support, helping them to more accurately understand the strengths and weaknesses of the current educational reality to obtain scientifically correct decisions. Data mining technology can analyze large and complex datasets efficiently and quickly and can find out the information hidden behind the data and the correlations, trends, and directions among the data [6]. If data mining technology is adopted in the education management system, it has important practical significance for the long-term development of education management. We are still continuing to study improved decision tree algorithms, and corresponding improvements have been made to the C4.5 algorithm from different perspectives. Among them are the time-consuming improvements for the C4.5 algorithm to process continuous attributes, using mathematical methods. The price is infinitesimal to improve the calculation efficiency of the information gain rate and so on [7].

The study conducted requirements analysis and detailed design of each functional module for this system and applied system functional structure diagram and functional use case diagram to describe the functional structure of the system and flow chart to illustrate the workflow of the system as a whole and in parts. The logical structure design, entity-relationship design, and physical model design were carried out for the database. Then the traditional ID3 algorithm was applied to the teaching quality assessment subsystem for analysis. To improve the efficiency of the system, the ID3 algorithm was optimized and improved on this basis to reduce the time complexity of its operation and improve the system operation efficiency. The goal of assessing and predicting teachers' teaching quality is achieved. This paper is divided into 5 sections, and the structure is arranged as follows: Section 2 discusses related work, the current state of research, and the application of classical algorithms, which lays the foundation for the research use in the subsequent chapters.  Section 3 is devoted to research on data mining-based decision support system for educational management.  Section 4 is devoted to results analysis.  Section 5 gives the conclusion. Data mining techniques are applied to educational management decision support system, relevant teacher information data, student information data, and social background information data are collected, and then the data are preprocessed; data mining algorithms are used to build models, test models, and apply models to help educators understand students' learning characteristics as much as possible, and the factors affecting teaching quality are analyzed. The value of the data related to students' learning information is maximized, and the role of educational data mining is truly brought into play.

## 2. Related Work

With the acceleration of campus digitalization in recent years, many universities have developed information management systems on their own or jointly with other universities to realize paperless offices and improve the scientific and high efficiency of daily work. The use of this system has accumulated a large amount of data, but colleges and universities have only made some inquiries, statistics, and reserves on these data and have not made full use of them. To make better use of these data and discover hidden valuable information, some universities have started to use data mining techniques to study, analyze, and solve problems. Therefore data mining technology will get more attention and application in the field of education [8]. Rodrigues et al. studied the application of data mining in the analysis of college entrance examination admission data through association rules and decision tree classification in the design of the college entrance examination information analysis system. Useful knowledge is mined for educational institutions, candidates, and colleges and universities [9]. Ghorbani and Ghousi conducted an in-depth study on educational management and data mining techniques in colleges and universities, using data mining related algorithms, and integrated a simple voting strategy through the study of simple improvement method of Apriori algorithm and ID3 algorithm, which has improved the educational management of students in colleges and universities and provided valuable guidance for students' training [10]. Farouk and Zhen used the educational technology of OLAM to analyze the education and admission of colleges and universities and identified the reasons and potentially useful information affecting students' enrolment through data mining techniques based on the educational data of previous years [11].

To pursue the rationality of educational decision-making and promote educational decision-making from experience to science, scholars at home and abroad have done a lot of research and discussion on educational decision support from different perspectives. Du et al. started the theoretical research in this area by proposing a data mining-based decision support system for educational management [12]. Shen et al. used the B/S model and analyzed the process dynamics of learners in the Moodle learning platform using network analysis techniques. They saved the learner dynamic record data in the management system to form real-time analytical models or statistical reports [13]. Operations such as classification patterns, clustering patterns, association rules, and other analytical methods as well as visual representations were formerly used, and the latter were used to represent common model generation from behavioural dynamic data. Lee et al. used data mining techniques to estimate the potential of the subjects [14]. Based on the organic integration of information about learners' metacognition, motivational goals, knowledge acquisition, and learning attitudes, they established models with learners as the objects and predicted the future development tendencies of learners, analyzed and improved educational method models, explored the effectiveness of various applications to learning systems, and established data-based computing models to improve and enhance effective learning of learners [15].

We use literature, academic paper statistics, and content analysis methods to systematically sort out and summarize the various literature related to educational data mining that has been published in China and abroad and then conduct an overall study and analysis to objectively analyze and compare the current research status of data mining technology in the field of education at home and abroad [16, 17]. The research trend of educational data mining in the future is discussed. Through the above literature, we can see that data mining is more oriented to the improvement of algorithms of data mining technology and the mining of existing education management data to analyze the intrinsic relationship between their different attribute values, but there is relatively little research on the valuable information that affects the relationship between new students' arrival rate and education management decisions. The purpose of this paper is to analyze and study educational management data from this perspective using mining techniques [11, 18]. The development of an educational management information management system plays an important role in promoting the development of higher education informatization. Use data mining algorithms to establish models, test models, and application models to help educators understand the characteristics of education management as much as possible, analyze the factors that affect teaching quality, and maximize the value of data related to education management information as much as possible [19]. Give full play to the due role of educational data mining [20].

## 3. Research on the Decision Support System for Education Management Based on Data Mining

### 3.1. Data Mining Education Model Construction

**Figure 1:**
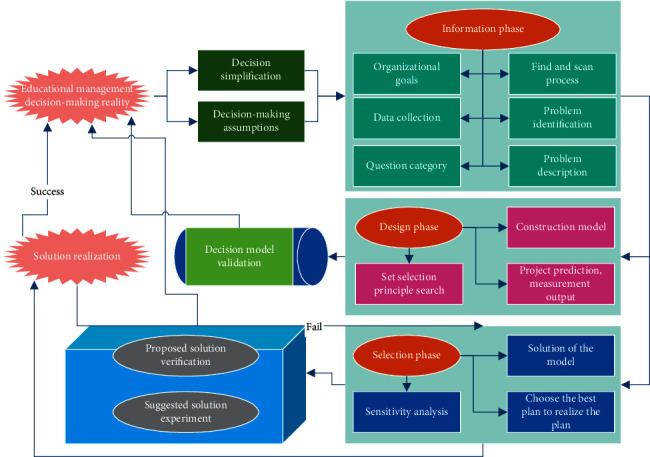
Educational management decision support process.

Reasonable goals in the decision-making process are a prerequisite for rational decision-making. The formation of the decision goal, the size of the goal, and the decision maker's understanding of the goal will affect the smooth implementation of the decision [21, 22]. In the process of establishing the goal, the nature, structure, and crux of the problem to be solved must be analyzed clearly before a reasonable decision target can be determined in a targeted manner. The decision goal must be very clear if the goal is too abstract or ambiguous, and ambiguous decision will be difficult to carry out, and the degree of achievement of the decision goal is also difficult to measure. The conceptual representation of the educational management decision support process is shown in Figure 1. The education management decision-making process mainly includes four basic stages: target determination, design plan, implementation plan, and evaluation plan. Each stage of the educational management decision-making process has a close relationship with the decision-making environment.

After the decision objectives are determined, a variety of possible options are further designed for decision-makers based on feasibility studies. The proposed feasible options require a combination of overall exhaustiveness and mutual exclusivity to avoid bias in the option selection process. Overall exhaustiveness means that the various options developed should include all possible options found. Mutual exclusivity means that only one option can be chosen among different options [23]. These mutually exclusive options should all be easy for decision makers to compare and choose. The analysis, evaluation, and comparison of options mean that the indicators of all options are compared including the technical, economic, and social environmental conditions, factors, and potential problems, and dynamic financial indicators related to the decision objective are compared and evaluated. The possible constraints and potential problems of each option, as well as preventive and emergency measures, are also compared and evaluated. The proper execution of the user in the data mining process can accelerate the execution of the data mining process. Providing an interactive interface provides users with easy operation, and the interactive interface conveys the generated results to the user in a timely and convenient manner, and the generated results can be diverse.

The task of data integration is to merge data from multiple data sources into a unified data store for data mining. Data sources may include multiple databases, data cubes, and data files. The main considerations of data integration are attribute matching, data redundancy, and the detection and handling of value conflicts [24]. For attribute matching, attribute merging is performed by examining the field meaning of each attribute, data type, and so forth. Redundant data is considered at two levels. For attribute-to-attribute redundancy, the meaning of attribute entities is mainly examined, and attributes are streamlined by retaining fine-grained metrics of attribute values and ignoring coarse-grained metrics. This is because coarse-grained attribute data can come from the transformation of fine-grained data. Some redundant relationships between attributes are difficult to detect and can be detected by detecting how much an attribute can contain an attribute, such as by calculating the Pearson coefficient, also called the correlation coefficient of two attributes; for example, the correlation coefficient *R* (*X*, *Y*) given two attributes *X* and *Y* is shown in equation (1), where N is the number of tuples, *x*_*k*_ and *y*_*k*_ are the values of *X* and *Y* in tuple *k*, respectively, $\overset{⟶}{X}$ and $\overset{⟶}{Y}$ are the means of *X* and *Y*, and *β* (*X*) and *β* (*Y*) are the standard deviations of *X* and *Y*. The ID3 algorithm is used to establish a decision tree model based on the attributes of the database samples. The algorithm requires more logarithmic operations, which results in time complexity and low operating efficiency. The same formula needs to be calculated repeatedly, so the algorithm still has room for improvement. The algorithm needs to be optimized.

(1) $$\begin{array}{c} R\left(X,Y\right)=\frac{\sum_{k=1}^{N}\left(x_{k}-\overset{⟶}{X}\right)*\left(y_{k}-\overset{⟶}{Y}\right)}{N*\beta \left(X\right)*\beta \left(Y\right)}. \end{array}$$

Facts are numerical measures, and multidimensional data models are organized around a fact, represented by a fact table that includes the name of the fact and the measure of the fact, as well as the code of each relevant dimension table [25]. A dimension is a pivot view or entity about which you want to keep records, and each dimension has a dimension table corresponding to it for data mining and decision analysis, which are Faculty Fact Table, Research Awards Fact Table, Research Results Fact Table, Research Funding Fact Table, Research Projects Fact Table, Talent Development Fact Table, Hardware Conditions Fact Table, and Subject Books Fact Table. Each fact table is associated with multiple dimension tables, such as discipline level, time, and unit level. The specific dimension tables are determined by the fact tables as shown in Table 1.

After obtaining the discrete dataset, we first train to obtain a complete decision tree using the ID3 algorithm, which works as follows: let data *D* be partitioned into a training set of class-labeled tuples, assuming that the class-labeled attributes have *N* different values, and *N* different classes *H*_*i*_ are defined (*i* = 1,…, *N*). Let *H*_*iX*_ be the set of tuples of class *H*_*i*_ in *X*, and let |*X*| and |*H*_*iX*_| be the numbers of tuples in *X* and *H*_*iX*_, as in equation (2), where *F* (*X*) is the average expected amount of information needed to classify the tuples in *X*; |*X*_*i*_|*∗F*(*X*_*i*_) represents the weights of the *i*-th division; *H*_*i*_ is the probability that any tuple in *X* belongs to class *H*_*i*_ and is estimated by |*X*_*i*_|/|*X*|.

(2) $$\begin{array}{c} \left\{\begin{array}{c} F\left(X\right)=\sum_{i=1}^{N}H_{ix}*\;\ln\left(X_{i}\right),\\ \overset{⟶}{F\left(X\right)}=\sum_{i=1}^{N}\frac{\left|X_{i}\right|*F\left(\left(X_{i}\right)\right)}{\left|X\right|}. \end{array}\right) \end{array}$$

The Bayesian posterior theory is used to test each branch of the decision tree, and the branches that are not considered to have sufficient generalization ability or are unreliable are removed from the tree, resulting in a more compact tree [26, 27]. The testing process is targeted at the knowledge of each rule translated from the decision tree, where each classification rule is obtained by searching from the root node of the decision tree top-down to a leaf node, and each classification rule consists of a conditional attribute tuple *X* and a conclusion classification label *H*_*x*_. For the test of each classification rule, we define two kinds of validation.

Adequacy verification:
  (3) $$\begin{array}{c} G\left(Hi\right)=\sum_{i=1}^{N}\frac{\left|H_{ix}\right|}{\left|X\right|}. \end{array}$$
Necessity validation:
  (4) $$\begin{array}{c} \left\{\begin{array}{c} G\left(H_{x}|Y\right)=\frac{\left|H_{x,X}\right|}{\left|X\right|},\\ G\left(H_{x}\right)=\frac{\left[H_{x}\right]}{\left|X\right|}, \end{array}\right) \end{array}$$

We need knowledge with clear interpretability yet do not want the classification and prediction process to be too demanding, so to facilitate the simplification of the processing problem, we draw on fuzzy theory and perform some fuzzy optimization of the decision rules in classification and prediction. The affiliation function for a certain attribute value *x* belonging to a certain interval (*G*_1_, *G*_2_] is established as

(5) $$\begin{array}{c} f\left(x\right)=\left\{\begin{array}{cc} 1-\frac{G_{1}-x}{G_{2}-G_{1}}, & x<G_{1},\\ 1,x=G_{1},x=G_{2}, & f\left(x\right)\subseteq \left[0,1\right],\\ 1-\frac{x-G_{2}}{G_{2}-G_{1}}, & x>G_{2}. \end{array}\right) \end{array}$$

A minimum acceptable value of affiliation *u* is specified, which can also be determined from a large number of experiments. In this paper, the value is set to *u* = 0.9 to simplify the determination process.

(6) $$\begin{array}{c} f\left(x\right)=\underset{i\subseteq \left[1,N\right]}{\max}\left(G_{1}-\left(1-u\right)\left(G_{2}-G_{1}\right),G_{2}+\left(1-u\right)\left(G_{2}-G_{1}\right)\right). \end{array}$$

The complexity of the educational system makes educational decision-making more difficult; an effective and rational educational decision support system will greatly contribute to the efficiency and rationality of educational decision-making. In this paper, we combine the characteristics of the educational decision-making process, the characteristics of the educational system itself, and the structural characteristics of the new generation decision support system to propose a decision support system model of educational management for the actual situation in the field of education and teaching.

### 3.2. Education Management Decision Support System Design Implementation

The data mining visualization system for education management information proposed in this study aims to provide decision support for education management information management in colleges and universities. The design goal of the system is to perform data mining on a large amount of education management data accumulated in the education management database over the years, and education management staff need to perform basic data analysis and data management on candidate data information through the online national college education management system. Finally, determine whether to admit or not and announce the admission results to the admitted candidates through the education management consulting system. The admitted candidates can also inquire about the situation of colleges and universities, previous years' education management admission score line, professional education management plan, application guidance, admission result, and issuance of admission notice through the education management information service system, while the relevant education management information managers of colleges and universities can also inquire about the historical information of education management and also use the data mining system to inquire about the application and admission and reporting situation through the system. Online analysis and visualization can be used to grasp education management information and make timely education management decisions.

The education management administration subsystem completes the function of maintaining information for education management records. All operations of the system require general records to be kept, and the general records are partially handled by the logs that participate in the shared section. The system starts when the user has successfully logged in and obtained the code that can control the scope of the system and expects to perform education management arrangement management operations. Education management arrangement includes 6 common functions of form presentation, adding, changing, deleting, finding, and detailing of education management teaching maintenance data. The administrator selects the hyperlink of education management and clicks on the specific list of each education management arrangement, and, with the help of clicking the function button given in the upper right corner, the system is commanded to complete the addition, deletion, and change of the list of activity plans.

**Figure 2:**
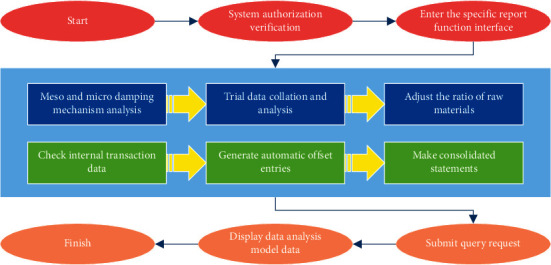
Flow chart of report query.

A large part of the data application uses the report query mode for data service, and so does the education management analysis module. The flow of the report query is briefly explained below, and its flow chart is shown in Figure 2. The first step of the report query is to log in to the system, at which time the system permissions are verified and only the functions and pages that the users are entitled to access are displayed; then the users enter the specific functional report interface according to their needs; the report interface functions are built based on various data analysis methods such as trend analysis, comparative analysis, cross-analysis, and retention analysis to establish the data model for query, and then the page is built based on the data model at the front end and the data organization at the back end. In the previous section, the detailed design and implementation of data storage and processing also mentioned that the data will be extracted from the dimension fields and indicator fields in the ADS layer, and now the query module can display the user-selectable dimensions based on the dimension field table. The user selects the dimension, the dimension value, and the data to send a query request, and the server calls the corresponding controller interface based on the requested URL and parameters to complete the query of the model data and return it to the Web client for display to the user.

The education management system designed and implemented in this paper uses a decision tree classification algorithm to generate a classification rule. One of the important tasks of the university's education management is to provide career guidance for students, so the education management system can use the information about students' schools to dig out their career direction. Therefore, the classification rules provided by the education management system for career guidance are as follows: Firstly, the students' school transcripts and comprehensive assessment are used as the input of the classification rules, and the courses taken by the students are classified as theoretical and practical courses; the spare time activities are classified as athletic and linguistic, and the students' roles in the activities are classified as leadership and nonleadership; based on this input information, the industry in which the students are employed is predicted. Finally, the employment information of previous students is used as an evaluation criterion. For example, grade analysis in university education management can obtain information about the knowledge points of students' grades in terms of question types and subject knowledge points of students' grades. It is very interesting to extract the important knowledge from the data warehouse of students' performance in higher education because there is a lot of information that can help to improve teaching management. The first step is to analyze the question types and the degree of correlation between question types, scores, knowledge points, and scores to summarize the strategies to train students.

## 4. Results and Analysis

### 4.1. Data Mining Education Model Analysis

**Figure 3:**
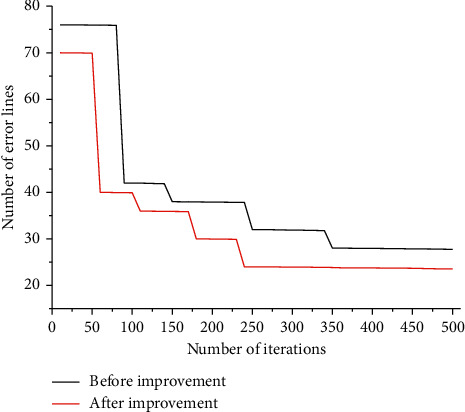
Convergence curves of the number of misclassified rows in the Vote dataset.

To test the effect of the improved algorithm, the objective function can be scaled up and processed, and the number of misclassified entries can be calculated directly when calculating the error rate. The improved algorithm has an obvious effect on datasets with large data volume and complex data types, so the comparison of the convergence effect on the Vote dataset is listed, as shown in Figure 3, for the convergence curves of the algorithm before and after the improved algorithm is carried out for 40 experiments and taken as the mean value. Through the experimental comparison, for the dataset with large data volume and complex data type, multiple experiments are conducted, and, within 500 iterations, the improved algorithm converges significantly faster than the unimproved one, is less likely to fall into the local optimal solution, and can converge to the global optimal value early.

**Figure 4:**
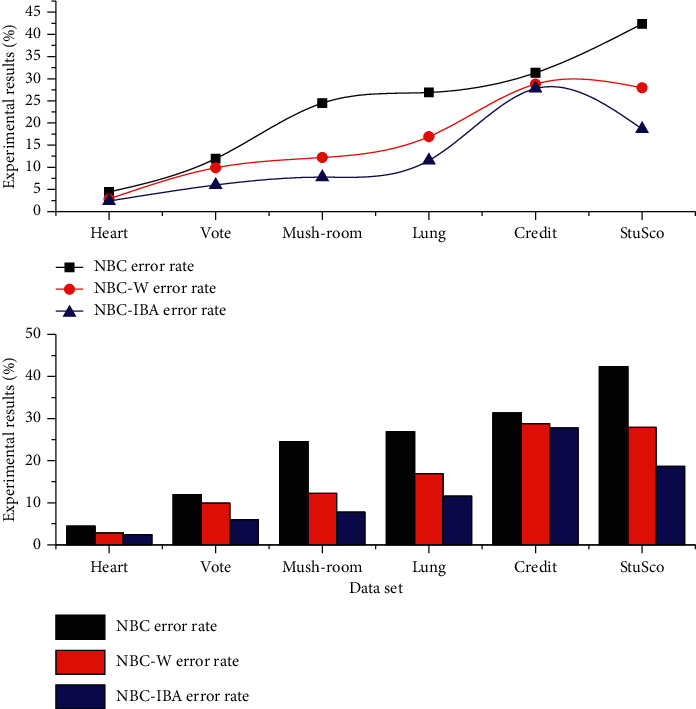
Comparison of classification error rates of three algorithms for six datasets.

Figure 4 shows the error rate of classification. When the proposed algorithm is used for classification, the error rates of all the datasets used are generally lower than those of NBC and NBC-W. If there is a dataset with a high classification error rate on the test data, it may be that the parameters set are not suitable, the training samples are not set reasonably well, or the data satisfy the assumption of conditional class independence. In general, NBC-IBA can classify accurately and reach the desired accuracy when dealing with most of the datasets. In terms of the running time of the algorithm, since the NBC-IBA algorithm needs to iteratively search for weights, the training time of the classifier is a little slower than that of NBC and NBC-W. After the training, not only is the speed comparable to that of NBC but also the accuracy rate is substantially improved in the subsequent classification process. In this chapter, the normalization criterion is applied when converting positions to weights, and the globally optimal positions are not simply used as weights.

**Figure 5:**
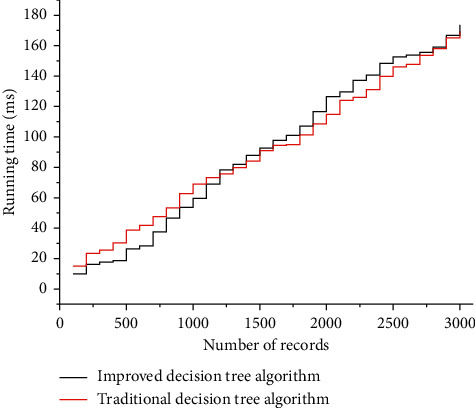
Running time.

Figure 5 shows the comparison of the time run using the 3000 datasets collected in the object, and after the above analysis is applied to the actual one, from the perspective of the overall operation process, the advantages of optimization are very obvious, which not only reduces the number of calculation, but also calculates the order of all nodes, reducing the time complexity of operation. The optimized decision algorithm converts the original logarithmic operation into a simple four-rule operation during the calculation of the information gain, say application formula, which greatly reduces the average and overall computational effort. Therefore, the improved decision algorithm reduces the efficiency without changing the overall effect of the original algorithm and can completely replace the old algorithm for application to the system.

### 4.2. Analysis of Educational Management Decision Support System

**Figure 6:**
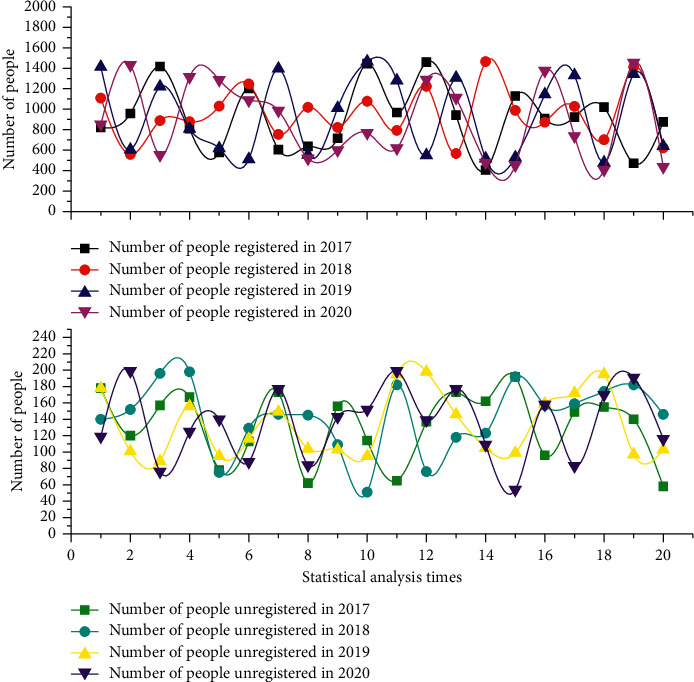
Comprehensive analysis.

Figure 6 shows the comprehensive analysis, which shows the basic information of 2017–2020, the students' reports of different majors, different places of origin, and different subjects, and the final educational decision can be made by combining the educational data of four years as follows. The system analysis module shows that, except for economic management and foreign languages, which have reached the education standard in the last three years, all other key majors have not been able to reach the basic number of education standards, so it is necessary to adjust the education number of majors in this area. The specialties of computer science, environmental engineering, and other science and technology majors can be adjusted appropriately according to the actual situation to make the specialties more rational and to get more students enrolled and admitted in this way. From the above conclusions, we can see that many factors are affecting the enrolment rate, and then we can use this as a guiding basis in our future educational work to help the college decision-makers to plan the opening of courses and the adjustment of the setting of majors, as well as to give some scientific guidance to the work of discipline management departments as well.

**Figure 7:**
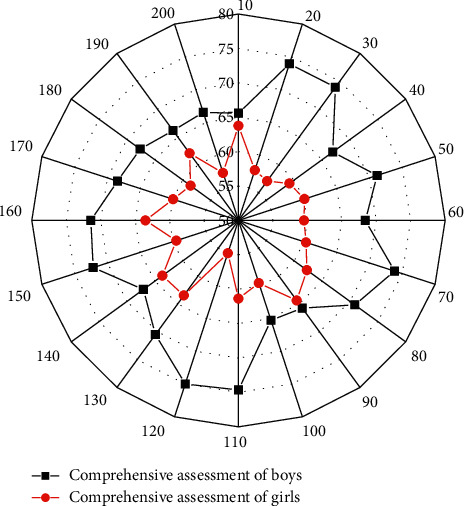
Correlation chart of comprehensive assessment scores.

Figure 7 further verifies that there is a significant difference in overall assessment scores between the preceding teacher-training category and the conductive category and between male and female students, while it can be known that teacher-training category is higher than the conductive categpry, and female students are higher than male students. In terms of comprehensive assessment, The students' comprehensive assessment in the academic year is linearly related to their intelligence, and ability has a significant impact on comprehensive assessment. There is a significant difference between male and female students, with an average score of 69.96 for female students, which is higher than the average score of 60.10 for male students; there is a significant difference between teacher training and knowledge, with an average score of 76.33 for teacher-training students, which is significantly higher than the average score of 55.28 for knowledge students. There is a significant relationship between students' employment and their overall assessment and also with gender.

**Figure 8:**
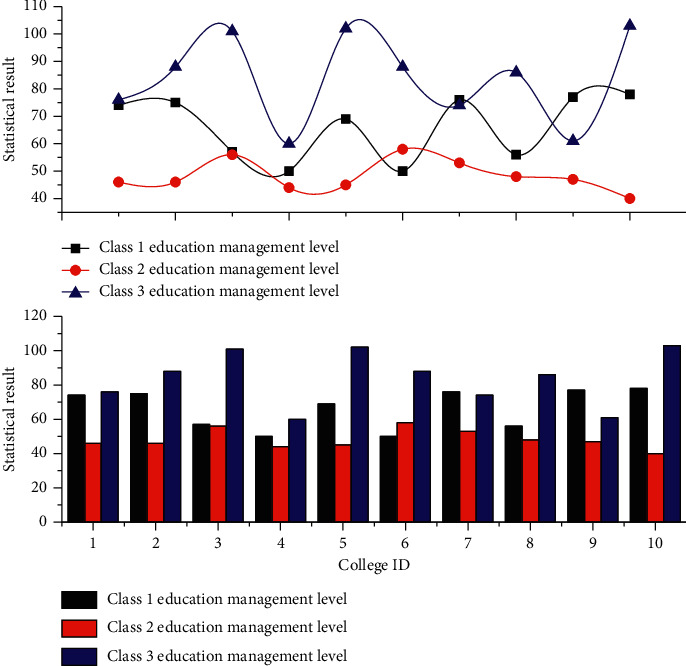
Statistical chart of educational management results by faculty.

The statistical analysis results of the whole school are used as an example of the display, as shown in Figure 8, which shows the statistical interface of the bar graph of education management results. The following statistical charts can be saved online. Figure 8 takes the statistical graph of education management of each college of the whole university as an example and shows the specific distribution of 3 types of education management levels of each college according to the college as a division unit, from which it can be seen which college is worthy of attention, which college is worthy of praise, and so on; the majors of a certain college, classes of a certain major, students of a certain class, and so forth are applicable to assist decision-makers in making decisions.

From the results of the above analysis, it is clear that there is a gap between male and female majors in educational technology in numerous aspects. To avoid this current gap phenomenon, the director of the program and the dean of instruction need to actively study and take favorable measures to guide male students to be competitive in academic life and other aspects to catch up with female students and narrow the gap. When setting the weighting coefficients for the comprehensive assessment of the student's academic year, the developers should focus on the coefficients for intellectual achievement and the criteria for ability bonus points. This is because these two parameters play a crucial role in the fairness and reasonableness of the comprehensive assessment of the student academic year. There is a need to continue to maintain the high weighting ratio of intellectual achievement in the comprehensive assessment. This will promote the all-around development of students' moral and intellectual and physical abilities while adhering to the principle that “students' primary responsibility is to learn” and giving full play to the effective means of reflecting students' learning in their intellectual performance. Professional teachers and administrators should gradually guide students to make efforts to study and stabilize their English grades from the time they enter the university, to achieve a relatively smooth passage of the fourth and sixth grades, avoiding the clinical learning style and the need to explore effective teaching methods to improve the English grades of male students. Although the results of comprehensive evaluation have an important impact on students' employment, students' single ability or special skills have a great advantage in employment. At the same time, in the employment process, the employment opportunities for men are higher than those for women, this through interviews and analysis of the employment situation in recent years, and the reason is mainly generated by the employment units in the treatment of gender differences between men and women and the point of conformity. Various activities are vigorously carried out to increase the opportunities for students to demonstrate their abilities and encourage them to take various certificate examinations to expand employment opportunities. Because of the current employment situation, female students should choose suitable majors according to gender characteristics when they are employed.

## 5. Conclusion

With the transformation of knowledge-based economy, the education industry is developing rapidly in this context, and modernization of education management is an important basis for promoting the sustainable development of the education industry, and an important symbol of modernization of education management is the construction of information system, which is an inevitable choice for the development of education modernization. In recent years, urbanization construction has been making breakthroughs, the population in need of education is relatively mobile, and the school layout has to be adjusted continuously as a result, which undoubtedly brings about serious problems to the education authorities, so it is extremely crucial to build an education management decision support system based on data mining. In this paper, the significance and background of the educational decision support management system are explained, and the user requirements, design scheme, and functional implementation are systematically described. The overall architecture and functions of the system are also analyzed, as well as the related system development environment and the required database design. The key technologies and difficulties involved in the development of the system are also discussed in detail. Finally, through testing the system, we found that the system can meet the user's requirements and can complete the basic education management work and has certain decision support functions. Of course, due to my limited ability, some functions of the system are not perfect, and the development of the system is still in the initial stage, so problems and shortcomings are inevitable, which are also the areas that need to be improved in the future. It is necessary to carry out research on the construction and application of various prediction models and monitoring models for the system prediction and dynamic monitoring mentioned in the discussion of the role of educational decision support system for evidence integration and benefit integration. These research skills will be a further extension and deepening of this research.

## Figures and Tables

**Table 1:** Fact and dimension tables for data mining data warehouse.

Serial number	Theme	Fact table	Dimension table 1	Dimension table 2	Dimension table 3
1	Scientific research awards	Scientific research award fact sheet	Unit dimension	Subject dimension	Time dimension
2	Scientific research results	Fact sheet of scientific research achievements	Subject dimension	Award type dimension	Unit dimension
3	Research funding	Research funding fact sheet	Award type dimension	Time dimension	Subject dimension
4	Research project	Research project fact sheet	Time dimension	Unit dimension	Subject dimension
5	Talent development	Talent training fact sheet	Unit dimension	Subject dimension	Award type dimension
6	Hardware condition	Hardware condition fact table	Subject dimension	Award type dimension	Time dimension

## Data Availability

The data used to support the findings of this study are available from the corresponding author upon request.
